# Perceptions of plastic pollution among inland fishery stakeholders in a subtropical reservoir

**DOI:** 10.1371/journal.pone.0353457

**Published:** 2026-07-09

**Authors:** Florence M. Murungweni, Manamela S. Mokata, Lubabalo Mofu, Jeffrey Lebepe, Samuel N. Motitsoe, Thendo Mutshekwa

**Affiliations:** 1 Department of Geography and Environmental Sciences, University of Venda, Thohoyandou, South Africa; 2 Department of Ichthyology and Fisheries Science, Rhodes University, Makhanda, South Africa; 3 South African Institute for Aquatic Biodiversity, Makhanda, South Africa; 4 Department of Biology and Environmental Science, Sefako Makgatho Health Sciences University, Ga-Rankuwa, Pretoria, South Africa; 5 School of Animal, Plant and Environmental Sciences, University of the Witwatersrand, Johannesburg, South Africa; 6 Department of Freshwater Invertebrates, Albany Museum, Makhanda, South Africa; 7 Institute for Water Research, Rhodes University, Makhanda (Grahamstown), South Africa; Manipal Academy of Higher Education (MAHE), INDIA

## Abstract

Plastic pollution is becoming a serious problem in freshwater ecosystems, impacting the livelihoods of people who rely on inland fisheries. Although plastic waste is widely discussed around the world, limited research has explored how local fishery stakeholders perceive pollution, particularly in Southern Africa. As such, using semi-structured interviews, the current study assessed the awareness, concerns, and solution perspectives of three different stakeholder groups, i.e., commercial fishers (CF), recreational fishers (RF), and fishmongers (FM) around Nandoni Dam, a subtropical reservoir in Limpopo Province, South Africa. Thirty participants, i.e., 10 per stakeholder, were interviewed, and our results showed that 96.7% of all stakeholders were aware of plastic pollution, yet 86.7% had limited understanding of microplastics. Perceptions of the impacts of plastic pollution varied across groups, with visitors (CF = 60%; RF = 60%; FM = 66.7%) and local residents (CF = 20%; RF = 20%; FM = 11.1%) being linked as a source of plastic pollution around Nandoni Dam. Willingness to participate in reducing plastic pollution was high across stakeholders (CF = 90%; RF = 90%; FM = 70%), with the majority emphasising the need for local municipal involvement and community engagement during clean-up activities and awareness initiatives. These findings highlight the need for targeted environmental education, and enhanced community-municipal collaboration to improve awareness and support collective action against plastic pollution in inland fisheries. Strengthening these actions could promote sustainable fisheries management, protect inland waters, and improve the well-being of the people who rely on these waters for food, income, and daily activities.

## Introduction

Plastics are strong, light, and flexible materials that are now a common part of our everyday life [1]. However, their non-degradable nature makes them one of the most persistent pollutants in the environment [2,3]. According to Kudzin et al. [4], global plastic production has risen sharply from about 2 million tonnes in 1950 to over 450 million tonnes by 2024. Although plastics have supported convenience and economic growth due to their low cost and wide use, poor disposal and mismanagement of plastic waste have led to serious environmental pollution [5,6]. Plastic bags, food wrappers, and other single-use plastics collectively, pose a significant environmental concern since they are broken down into very small particles that get easily transported into waterways through runoff [7]. Once in aquatic environments,these particles get further broken down into microplastics (<5 mm), which then gets ingested by aquatic organisms and subsequently transferred through the aquatic food web [8,9a].

As such, plastic pollution is declared a global threat for freshwater and marine ecosystems, with an estimated 19–23 million tons of plastic waste entering rivers, lakes, and oceans each year [10]. For example, plastic particles ingestion in fish has shown to have serious physiological effects that leads to interval injuries, reduced reproduction, growth and high population mortality [11, 12]. Plastics can also release or absorb harmful chemicals that compromise aquatic health and biodiversity [13,14]. Since fish serve as a vital source of food and income for millions of people, plastic pollution not only threatens aquatic biodiversity but also human health and livelihoods [12,15]. This growing concern is particularly critical for communities that depend directly on inland fisheries, where pollution can undermine both ecosystem integrity and local economies [16].

Inland fisheries provide critical economic, nutritional, and social benefits to communities in developing countries [16,17]. However, freshwater systems are increasingly vulnerable to pollution from urbanization, agriculture, and plastic waste [18–20]. In inland areas, fishers and fishmongers rely directly on these ecosystems for their livelihoods, yet they also contribute to plastic waste through the use and disposal of fishing gear, packaging, and other plastic materials [21]. Abandoned or discarded fishing gear can entangle aquatic organisms, cause injuries, or reduce fish availability, threatening both ecological and economic sustainability [22,23]. Consequently, plastic pollution affects not only ecosystem functioning but also fisheries productivity and the food security for local communities that rely on these resources.

Globally, several measures have been introduced to mitigate plastic pollution, including bans on single-use plastics, recycling initiatives, and improved waste management systems [24]. For example, countries such as Canada and France have enacted national bans and phase-out programs targeting single-use plastic bags, cutlery, and packaging. In contrast, the United States has banned microbeads in cosmetics and introduced various state-level restrictions on disposable plastic items [25,26]. Similarly, the United Nations Environment Programme (UNEP) has established international frameworks to address plastic pollution through life cycle management and policy coordination [27,28]. In South Africa, initiatives such as plastic bag levies, waste facility licensing, and public awareness campaigns led by organizations such as Plastics SA have been implemented to encourage responsible plastic use and recycling [29].

Despite growing awareness of plastic pollution globally, most social-based research has concentrated on marine environments [e.g., 22,30,31] with limited attention given to freshwater ecosystems and inland fisheries [32], particularly in Southern Africa. Understanding local perceptions and awareness of plastic pollution among those directly dependent on these ecosystems is crucial for identifying knowledge gaps and designing effective interventions [33]. Fishers and fishmongers play a vital role in sustaining livelihoods and food security for local communities, yet their practices and perceptions can either help reduce or worsen plastic pollution in freshwater ecosystems that communities depend on. As such, this study aims to (*i*) assess the perceptions, concerns, and solutions regarding plastic pollution among three key stakeholders, namely commercial fishers, recreational fishers, and fishmongers in a subtropical freshwater fishery of South Africa; and (*ii*) examine the socio-demographic and contextual factors influencing their understanding and experiences of plastic pollution. By investigating these aspects, this study seek to understand how social perceptions influence environmental behaviour, providing evidence to inform and guide targeted education programs, policy actions, and community-based management strategies for mitigating plastic pollution in freshwater fisheries.

## Methods and materials

### Study area

This study was conducted in the subtropical region of South Africa around Nandoni Dam (22°59′11″S, 30°36′16.19″E) about 10 km from Thohoyandou in the Vhembe District, Limpopo Province (Table 1). The surveyed participants were located in the villages of Mulenzhe, Mutoti and Dididi, each with a population of approximately 3,000. Nandoni Dam is a 47 m high earth-fill reservoir and has a 200 m spillway. The dam has a catchment area of 1380 km², a length of 2215 m, and a storage capacity of ~16.4 million m³ [34]. Built as a government water scheme, it supplies water for domestic use, irrigation, forestry, and supports a small commercial fishery [34]. The dam is primarily fed by the Luvuvhu River and its tributaries, the Mutshindudi and Mutale rivers, which pass through farmland and landfill areas [34]. It provides water, irrigation, and fishing opportunities for local communities and attracts visitors for recreational fishing through its camping and lodging facilities [34]. The region experiences warm, humid summers with an average temperature of 23 °C, cool, dry winters averaging 17 °C, annual rainfall ranging from 610 to 800 mm, and an average yearly runoff of approximately 519 million m³ (https://www.weathersa.co.za/).

**Table 1:** Coordinates of selected survey location around the subtropical reservoir of Nandoni Dam and their coordinates.

Villages	Coordinates
Mulenzhe	23°00’02.7“S 30°33’43.0”E
Mutoti	22°57’53.3“S 30°35’49.6”E
Dididi	23°00’41.7“S 30°30’31.0”E

### Ethical clearance and participant consent

The research protocol was reviewed and approved by the Research Committee of the Faculty of Science, Engineering and Agriculture at the University of Venda, in accordance with the University of Venda Policy on Research Ethics (2022) and the Protection of Personal Information Act, 2013 (Act No. 4 of 2013) (https://www.univen.ac.za/research/research-ethics/) (Ethical approval ref: FSEA/24/GES/10). This approval included data collection from inland fishery stakeholders around Nandoni Dam and confirmed that the study complied with ethical standards, with minimal risk to humans, animals, and the environment (Category 1). Informed consent was obtained from all participating stakeholders.

Before each interview, the purpose of the study was clearly explained to participants, and verbal informed consent was obtained from all participants prior to their participation following a similar approach to that employed by Wootton et al. [22]. The interviewer documented each participants verbal consent in accordance with the procedures approved by the ethics committee. Participants were also given an opportunity to ask questions and were informed about the nature, objectives, potential risks, and benefits of the study before agreeing to participate. Participants were assured that their information would remain private and confidential, following two key principles: (*1*) secrecy and (*2*) anonymity [35]. Participation in the study was entirely voluntary, and participants were free to withdraw at any time without penalty [35]. They were also informed that they could skip any question they did not wish to answer. No personal identifying information was collected, and all responses were anonymised by assigning a unique number to each questionnaire.

### Study design and participants

The study was conducted over a four-month period from 03/05/2025 to 09/08/2025 using a qualitative research design that employed semi-structured interviews to explore the perceptions, impacts, and proposed solutions to plastic pollution among key stakeholders, i.e., commercial fishers (CF), recreational fishers (RF), and fishmongers (FM), around Nandoni Dam, following a similar study by Wootton et al. [22] in coastal waters of the state of South Australia. These stakeholder groups were selected because Nandoni Dam supports subsistence and small-scale fisheries as well as recreational angling activities, with commercial fishers and fishmongers forming part of the local fish value chain and recreational fishers regularly using the dam for leisure fishing. As primary users and dependents of the dams aquatic resources, these groups are directly exposed to and impacted by plastic pollution within this freshwater ecosystem, making them appropriate stakeholders for the present study. The qualitative approach was selected for its strength in capturing in-depth views, experiences, and motivations of participants regarding environmental issues within their local context [36].

Participants were recruited through a combination of purposive and snowball sampling, ensuring representation across different stakeholders [37]. Recruitment was facilitated through direct engagement at fishing sites and local markets around Nandoni Dam. The study reached data saturation through iterative assessment of thematic saturation within each stakeholder group, whereby interviews were continued until no new codes or themes emerged across successive interviews, specifically after at least two consecutive participants per group. This process resulted in a total sample of 30 participants (10 participants × 3 stakeholder groups). Saturation was assessed through concurrent coding during data collection, with interview transcripts continuously analysed to determine whether additional data contributed new insights or whether responses had become repetitive across emerging themes.

### Data collection

Semi-structured interviews were guided by a structured interview schedule developed using Wootton et al. (2022) as a thematic framework and Mabadahanye et al. [38] for socio-demographic variables and subsequently adapted to align with the objectives and local context of the present study. The interview guide was structured around five core thematic areas derived from Wootton et al. [22], including (1) socio-demographic information, (2) fishing or trading background, (3) awareness and understanding of plastic pollution, (4) concerns and perceived impacts, and (5) possible solutions (S1 Table). All questions were revised and contextualised. No core thematic areas were removed, but wording and probes were adjusted for contextual relevance. To ensure clarity and accessibility, the interview questions were administered orally in the participants native language, with real-time interpretation where necessary, and participants were encouraged to respond in their preferred language. No formal written translation was required as interviews were conducted verbally in the field. Each interview lasted approximately 15–30 minutes, and answers were recorded on a mobile phone (with permission) to ensure all information was captured correctly for later analysis.

### Data analysis

The data obtained from the survey were analysed using Microsoft Excel (Windows 11) and IBM SPSS version 25. Microsoft Excel was used to organise, code, and manage the qualitative responses. IBM SPSS was used to generate descriptive statistics for the quantitative components of the survey, including participant demographics and the frequency of response patterns. A thematic analysis approach was applied, whereby interview transcripts were systematically read and re-read to familiarise with the data. Initial codes were developed deductively based on the interview guide and study questions and then refined inductively as patterns and recurring ideas emerged across responses. These codes were subsequently grouped into broader themes corresponding to the study objectives (as summarised in S1 Table). Each response was assigned a unique identifier to maintain traceability and anonymity. For example, “CF2/T3/Q2” denotes Commercial Fisher Participant 2, Theme 3, Question 2.

## Results

### Socio-demographic and background

The results indicated that most participants in the commercial fisher (CF) category were male (80%, *n* = 8), with females representing 20% (*n* = 2). Similarly, recreational fishers (RF) were predominantly male (70%, *n* = 7), while females accounted for 30% (*n* = 3). In contrast, fishmongers (FM) showed an equal gender distribution, with males and females each representing 50% (*n* = 5) (Table 2). Most participants were between 31 and 40 years, mainly commercial fishers (70%, *n* = 7) and fishmongers (60%, *n* = 6), while recreational fishers were mostly 18–20 years (40%, *n* = 4). The 21–30 years group included a few participants from all categories, except for fishmongers, who were predominantly 41–50 years old (30%, *n* = 3) (Table 2). Regarding education, participants who had only completed high school were the most common (40–60% across groups), followed by those with diplomas (20–40%), certifications (10–20%), and degrees, which were more prevalent among recreational fishers (20%). A small proportion of commercial fishers and fishmongers were uneducated (10% each) (Table 2). Years of experience in fishing or fish trading ranged from 1 to 20 years across stakeholders. The fish species commonly caught in Nandoni Dam are provided in S2 Table.

**Table 2:** Socio-demographic characteristics of participants around Nandoni Dam. Abbreviation: CF – Commercial fisher, RF – Recreational fisher, FM – Fishmongers.

Variable	CF	RF	FM
Gender			
Male	8(80%)	7 (70%)	5 (50%)
Female	2 (20%)	3 (30%)	5 (50%)
Age group			
18-20	0 (0%)	4 (40%)	0 (0%)
21-30	3 (30%)	3 (30%)	1 (10%)
31-40	7 (70%)	3 (30%)	6 (60%)
41-50	0 (0%)	0 (0%)	3 (30%)
Education level			
High school	6 (60%)	4 (40%)	5 (50%)
Uneducated	1 (10%)	0 (0%)	1 (10%)
Diploma	2 (20%)	4 (40%)	2 (20%)
Degree	0 (0%)	2 (20%)	0 (0%)
Certification	1 (10%)	0 (0%)	2 (20%)

### Awareness and perceptions of plastic pollution

**Fig 1:**
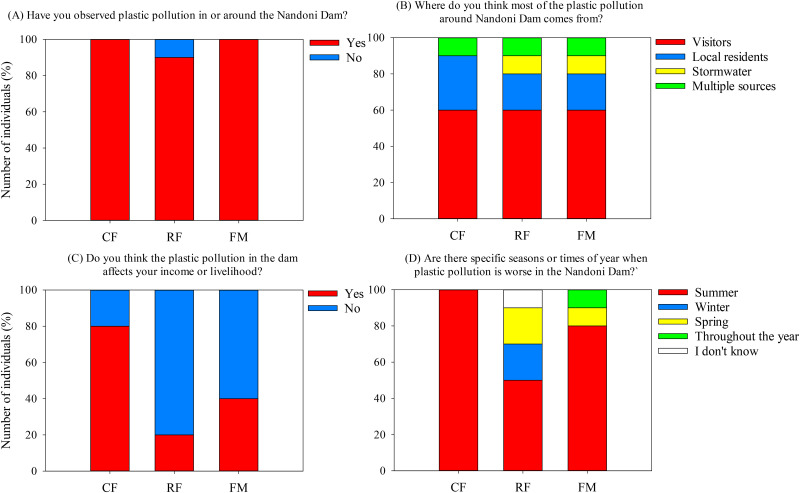
Local fishery stakeholders’ perception and awareness (%) regarding plastic pollution around Nandoni Dam. Abbreviation: CF – Commercial fisher, RF – Recreational fisher, FM – Fishmongers.

**Fig 2:**
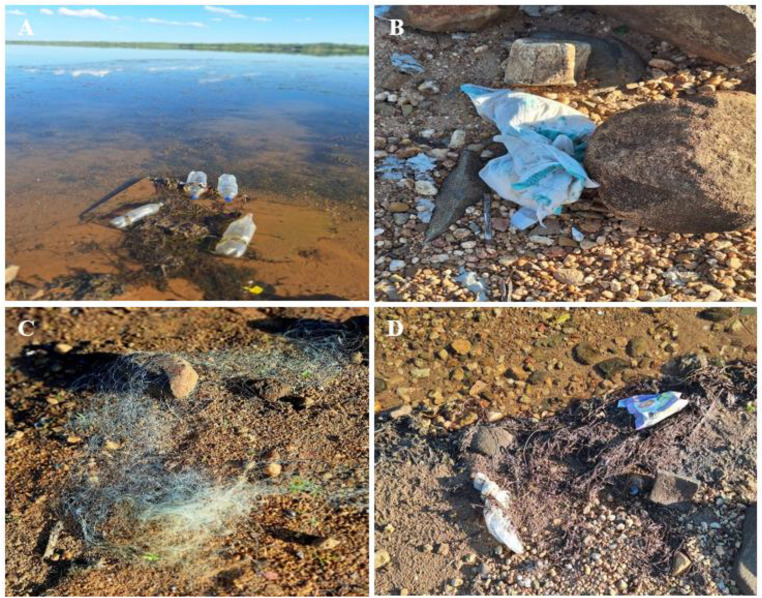
Plastic pollution observed around Nandoni Dam during data collection, including (A) plastic bottles, (B) diapers, (C) fishing nets, and (D) plastic bags and detergent wrappers. Photographs taken by Mokana Sonty Manamela during field sampling.

The results indicated that most participants have observed plastic pollution around Nandoni Dam (Fig 1A; S5 Table). Participants described plastic pollution as “*an increase of plastic in the environment*” (CF1T3Q7), “*the throwing of plastic everywhere*” (FM2T3Q7), and “*when plastic is thrown into the environment, harming animals*” (RF3T3Q7). Most participants (96.7%, *n* = 29) reported observing plastic pollution in or around the Nandoni Dam, while only 3.3% (*n* = 1) had not. Participants noted that plastic pollution mostly originates from “*local residents near the dam and upstream communities*” and “*visitors or tourists*” (Fig 1B; S3 Table). The most observed plastic items by participants included plastic bottles (56.7%, *n* = 17), plastic bags (40.0%, *n* = 12), and diapers (30.0%, *n* = 9) (Fig 2). Participants further showed a lack of awareness of microplastics, with only 10% (*n* = 3) familiar with the term and 90% (*n* = 27) unaware across all groups.

### Perceived impacts of plastic pollution

All participants (100%) reported that they have never experienced health issues related to eating fish from Nandoni Dam. When asked whether plastic pollution affects income or livelihoods, the responses diverged among stakeholders. Most commercial fishers believed it negatively impacts income (“*when there is plastic, fish die and our business is affected*” – C9T4Q16; “*it affects our catch, therefore our income*” – C1T4Q16) (Fig 1C; S5 Table). When asked whether they had observed any changes in fish availability over the years due to increasing plastic pollution, responses were mixed. Participants reported mixed views regarding seasonal variation in plastic pollution. Commercial fishers (100%, *n* = 10) and fishmongers (80%, *n* = 8) reported higher plastic pollution in summer, while recreational fishers reported various seasons (Fig 1D; S4 Table).

### Willingness to participate

**Fig 3:**
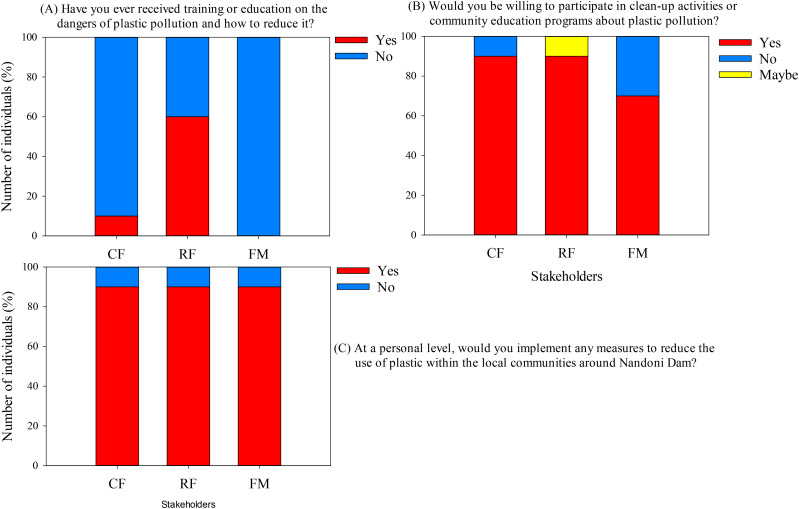
Local fishery stakeholders concerns and solutions (%) regarding plastic pollution around Nandoni Dam. Abbreviation: CF – Commercial fisher, RF – Recreational fisher, FM – Fishmongers.

Most recreational fishers (80%, *n* = 8) and fishmongers (90%, *n* = 9) reported that they had not received training regarding plastic mitigation (Fig 3A; S5 Table). Willingness to participate in clean-up activities or community education programs was generally high. 26 of 30 participants (86.7%) responded “*Yes*”. One recreational fisher (10%, *n* = 1) responded “*Maybe*” and among fishmongers, 3 participants (30%) declined (Fig 3B). When asked about solutions to reduce plastic pollution around Nandoni Dam, most participants (70%; *n* = 21) suggested that the local municipality increase the number of waste bins and implement community awareness campaigns. When asked about their willingness to act to reduce plastic pollution, nearly all participants said they would act personally (Fig 3C), such as “*placing bins*” (CF1T5Q23) or “*collecting plastics*” (CF7T5Q23). Only a few fishmongers were reluctant, “*I will only implement measures if I get paid*” (FM1T5Q23).

### Influence of socio-demographic characteristics on behavioural factors

Thematic analysis of socio-demographic characteristics and behavioural responses indicated that relationships between variables were generally weak and inconsistent across participants. Overall, no clear or consistent patterns were observed linking education level, gender, or age group with awareness of plastic pollution or related behavioural responses. Participants with higher education levels did not consistently demonstrate greater awareness of plastic pollution, knowledge of microplastics, or stronger willingness to participate in environmental campaigns. Similarly, perceptions of income-related impacts and observed pollution levels varied widely across educational backgrounds, with no clear directional trend emerging from the data. Gender and age group also did not show consistent differences in awareness or behavioural intentions. Responses related to observed plastic pollution, microplastic knowledge, and willingness to engage in environmental action were distributed across all demographic categories without clear pattern.

## Discussion

Plastic pollution is increasingly recognised as a major environmental challenge, threatening freshwater biodiversity, fisheries productivity, and human livelihoods [21,39]. In developing countries like South Africa, the impacts of plastic pollution are particularly severe, in rural areas, disrupting local livelihoods, food security, and ecosystem health [40]. The current study focuses on the local freshwater fishery in Nandoni Dam, assessing how local inland fishery stakeholders, i.e., commercial fishers, recreational fishers, and fishmongers, perceive and respond to plastic pollution. Across all stakeholders, awareness of general plastic waste was high, but knowledge of tiny plastics (i.e., microplastics) and their ecological impacts was limited. Participants demonstrated a strong willingness to engage in clean-up initiatives and community education yet relied heavily on Thulamela Local Municipality to implement solutions.

Socio-demographic characteristics did not show a consistent or systematic influence on environmental awareness or behavioural engagement. The predominance of male participants, particularly among commercial fishers and fishmongers, reflected the gendered nature of fishing-related activities, where men are more commonly involved in harvesting and trading fish [41,42]. Most participants only had a secondary education, particularly among commercial fishers and fishmongers. This is not surprising given the limited employment opportunities in South Africa [43,44], where people in rural areas with lower educational levels often rely on informal livelihood activities such as fishing. This is consistent with findings from Mabadahanye et al. [38], and Mutesasira and Marongwe [45], who reported that experiential knowledge often outweighs formal education in shaping environmental perceptions in rural South African communities. Younger adults, mainly recreational fishers, demonstrated greater responsiveness to environmental issues, supporting the Environmental Literacy framework [46], which suggests that younger generations tend to display greater environmental concern due to broader access to education and digital information [47].

Awareness and concerns regarding plastic pollution varied between stakeholder groups, reflecting their differing interactions with the aquatic environment, consistent with findings by Guerra-Marrero et al. [48] and Wootton et al. [22]. Commercial fishers in our current study were aware of visible plastic pollution, mainly bottles, bags, and diapers, but many perceived it as a manageable issue, citing informal clean-up efforts as evidence of local improvement. Recreational fishers, on the other hand, were more vocal about pollution caused by visitors and local residents, frequently shifting responsibility away from themselves. This tendency to shift responsibility is similar to findings by Rohrschneider [49] and Grušovnik [50], where people often blame others for environmental problems. This was also seen in our study, where some participants showed limited personal responsibility, for example saying they would only take action if they were paid (“*I would only implement measures if I get paid*” (FM1T5Q23)), and by suggesting that municipalities should be mainly responsible for managing plastic pollution.

Fishmongers primarily focused on selling fish and keeping their market areas visibly clean to attract customers, rather than showing concern about plastic pollution in Nandoni Dam and its potential effects on fish and consumers. This pattern is consistent with finding by Wootton et al. [22], who observed that fishmongers often prioritised market presentation and sales over broader environmental management issues and customer health implications. Overall, the findings indicated that perceptions of plastic pollution are largely influenced by visibility, with participants easily identifying larger debris but rarely recognising tiny contamination in water or in fish. The results also revealed a limited understanding of how larger plastics break down into tiny particles, such as microplastics, which can be ingested by fish and accumulate in their bodies [12]. As noted by Azevedo-Santos et al. [39], there is a need for targeted awareness and education initiatives in local communities to help enhance plastic pollution awareness and understand their potential impacts on aquatic life and human health.

While most participants had never received any form of environmental training, their stated willingness to participate in community clean-ups and environmental awareness programmes was high, indicating a strong foundation for future community-based interventions. However, despite this reliance on formal authorities, both commercial and recreational fishers expressed a willingness to actively participate in clean-up campaigns and community awareness programmes. This willingness is partly motivated by a desire to maintain access to fishing resources, as some fishers noted that failure to address environmental concerns could lead local authorities, NGOs, or community groups might impose restrictions or even ban fishing activities in affected water bodies. Additionally, fishers recognized that proactive engagement in pollution mitigation could help safeguard the long-term health of aquatic ecosystems, which are critical for sustaining their livelihoods and recreational activities. While participants demonstrated awareness of environmental impacts, recreational fishers indicated their continued reliance on fishing nets due to their low cost and ability to catch large quantities of fish quickly, highlighting a tension between immediate economic benefits and long-term sustainability. Participants also indicated an interest in collaborating with local schools, community organizations, and environmental groups to raise awareness about plastic pollution.

Overall, several challenges emerged from participants across all stakeholders. Participants frequently mentioned the absence of waste bins, inadequate maintenance, and the lack of enforcement of disposal regulations in their community. Enhancing waste management infrastructure in communities around Nandoni Dam, including the provision of bins and local enforcement measures, could significantly reduce plastic littering and input into Nandoni Dam [34]. Additionally, dependence on plastic packaging for fish trading remains a challenge. Similar findings were found by Kumar et al. [51], who highlighted the difficulties of replacing plastic in perishable goods markets without compromising quality or cost.

Future research should prioritise long-term monitoring of both visible plastics and microplastic contamination in Nandoni Dam [e.g., 52] and comparable freshwater systems such as natural springs [53]. Investigating seasonal variability, pollutant sources, and bioaccumulation in key fish species using Nandoni Dam as a case study would provide critical insights for ecological risk assessments and fisheries management. From a practical perspective, interventions should combine targeted environmental education with improvements to waste management infrastructure, such as the provision of bins, enforcement of disposal regulations, and promotion of alternatives to single-use plastic in fish trading. Strengthening participatory governance frameworks, involving commercial fishers, recreational fishers, fishmongers, and local authorities, could enhance accountability and foster sustainable behavioural change. These strategies would support both environmental conservation and the socio-economic resilience of communities dependent on freshwater fisheries, providing a foundation for adaptive management and policy development across South Africa and the broader Southern African region.

Despite providing valuable insights, this study has several limitations that should be considered when interpreting the results. The participant sample was predominantly male and comprised commercial and recreational fishers and fishmongers, which may limit the generalisability of findings across all stakeholder groups, including women and other community members [54]. Data collection occurred from May to August, potentially overlooking seasonal variations in plastic pollution and fishing activity. Additionally, the study relied on self-reported perceptions, which may be influenced by recall bias or social desirability [55]. Finally, the research was confined to Nandoni Dam, and no direct measurements of waste management infrastructure or municipal enforcement were conducted, restricting broader inferences about governance effectiveness and environmental conditions in other freshwater systems.

## Conclusion

This study reveals that stakeholders around Nandoni Dam are aware of visible plastic waste but have a limited understanding of tiny plastics and their risks. Perceptions differed among stakeholders, with commercial fishers concerned about impacts on catches, recreational fishers often blaming external sources, and fishmongers focusing mainly on market cleanliness. Although most participants were willing to join clean-up and awareness activities, their efforts are limited by poor waste management infrastructure and a strong reliance on the government to address pollution. Improving community waste services, increasing local environmental education, and involving fishers and fishmongers in co-managed clean-up and monitoring initiatives could strengthen local action. Future work should include seasonal monitoring of plastic pollution and promote affordable alternatives to plastic fishing gear.

## Supporting information

S1 Table Interview questions different stakeholders. Please note that this guide represents the main themes covered in the semi-structured interviews. It does not include additional prompts or general, non-leading follow-up questions. Minor variations were made depending on the participant stakeholder group.
(DOCX)

S2 Table Table of common and scientific names of fish species caught in Nandoni Dam. This list reflects only the fish reported by participants during data collection and does not represent all species found in the dam.
(DOCX)

S3 Table Local fishery stakeholder responses (%) regarding sources of plastic pollution around Nandoni Dam. Abbreviation: CF – Commercial fisher, RF – Recreational fisher, FM – Fishmongers.
(DOCX)

S4 Table Local fishery stakeholder responses (%) regarding seasonal occurrence of plastic pollution around Nandoni Dam. Abbreviation: CF – Commercial fisher, RF – Recreational fisher, FM – Fishmongers.
(DOCX)

S5 Table Local fishery stakeholder responses (%) to multiple questions. Abbreviation: CF – Commercial fisher, RF – Recreational fisher, FM – Fishmongers.
(DOCX)
