# Supplementary material for: Perceptions of plastic pollution among inland fishery stakeholders in a subtropical reservoir
Source: PLoS One. 2026 Jul 9;21(7):e0353457. doi: 10.1371/journal.pone.0353457 (PMC13349089; doi:10.1371/journal.pone.0353457)
Supplement: S1 Table — Please note that this guide represents the main themes covered in the semi-structured interviews. It does not include additional prompts or general, non-leading follow-up questions. Minor variations were made depending on the participant stakeholder group. (DOCX) [file pone.0353457.s001.docx]

**S1 Table**: Interview questions different stakeholders. Please note that this guide represents the main themes covered in the semi-structured interviews. It does not include additional prompts or general, non-leading follow-up questions. Minor variations were made depending on the participant stakeholder group.

| **Theme 1: Sociodemographic** |
| --- |
| 1. *Respondent type:* 2. *Gender:* 3. *Educational level* 4. *Age group:* |
| **Theme 2: Background Information** |
| 1. *Tell me a bit about yourself? (tailor to specific person) How long have you been working as a commercial fisher/fishmonger/recreational fisher?* 2. *Which fish species do you commonly catch/trade, and have you noticed plastic debris in or around these catches* |
| **Thème 3: Perceptions on Plastic Pollution** |
| 1. *What do you understand by the term “plastic pollution”?* 2. *Have you observed plastic pollution in or around the Nandoni Dam?* 3. *Where do you think most of the plastic pollution in the Nandoni Dam comes from?* 4. *What types of plastic have you seen?* 5. *Do you think plastic pollution in the dam is increasing, decreasing, or staying the same? Why?* 6. *Have you ever heard of the term “microplastics”?* |
| **Theme 4: Concerns and Impacts** |
| 1. *Have you or someone you know experienced health issues you think may be linked to eating fish from the Nandoni Dam?* 2. *Have you noticed any changes in the availability or health of fish in the Nandoni Dam over time?* 3. *Are there specific seasons or times of year when plastic pollution is worse in the Nandoni Dam?`* 4. *Do you think the plastic pollution in the dam affects your income or livelihood?* |
| **Theme 5: Solutions** |
| 1. *Are there any clean-up efforts or plastic pollution awareness campaigns currently taking place in your area?* 2. *Have you ever received training or education on the dangers of plastic pollution and how to reduce it?* 3. *Would you be willing to participate in clean-up activities or community education programs about plastic pollution?* 4. *Which of the following do you think would help most in reducing plastic pollution?* 5. *Who do you think should be responsible for keeping the Nandoni Dam clean?* 6. What types of measures would you suggest as possible solutions to address the impact of plastic pollution and microplastics in fish? 7. At a personal level, would you implement any measures to reduce the use of plastic within the local communities around Nandoni Dam? |
