# Supplementary material for: Perceptions of plastic pollution among inland fishery stakeholders in a subtropical reservoir
Source: PLoS One. 2026 Jul 9;21(7):e0353457. doi: 10.1371/journal.pone.0353457 (PMC13349089; doi:10.1371/journal.pone.0353457)
Supplement: S2 Table — This list reflects only the fish reported by participants during data collection and does not represent all species found in the dam. (DOCX) [file pone.0353457.s002.docx]

**S2 Table**: Table of common and scientific names of fish species caught in Nandoni Dam. This list reflects only the fish reported by participants during data collection and does not represent all species found in the dam.

| Common name | Scientific name |
| --- | --- |
| Banded tilapia | *Tilapia sparmanni* |
| Bowstripe Barb | *Barbus viviparus* |
| largemouth bass | *Micropterus salmoides* |
| Leaden Labeo | *Labeo molybdinus* |
| Lowveld Largescale Yellowfish | *Labeobarbus marequensis* |
| Mozambique tilapia | *Oreochromis mossambicus* |
| Redbreast tilapia | *Tilapia rendalli* |
| Redeye labeo | *Labeo cylindricus* |
| Sharptooth Catfish | *Clarias gariepinus* |
| Threespot Barb | *Barbus trimaculatus* |
