# Supplementary material for: Perceptions of plastic pollution among inland fishery stakeholders in a subtropical reservoir
Source: PLoS One. 2026 Jul 9;21(7):e0353457. doi: 10.1371/journal.pone.0353457 (PMC13349089; doi:10.1371/journal.pone.0353457)
Supplement: S3 Table — Abbreviation: CF – Commercial fisher, RF – Recreational fisher, FM – Fishmongers. (DOCX) [file pone.0353457.s003.docx]

**S3 Table**: Local fishery stakeholder responses (%) regarding sources of plastic pollution around Nandoni Dam. Abbreviation: CF – Commercial fisher, RF – Recreational fisher, FM – Fishmongers.

| Sources of plastic pollution | CF | RF | FM |
| --- | --- | --- | --- |
| Visitors or tourists | 60 | 60 | 66.7 |
| Local settlements / upstream communities | 20 | 20 | 11.1 |
| Stormwater/rivers feeding dam | 0 | 10 | 11.1 |
| Multiple sources (combined mentions) | 20 | 10 | 11.1 |
