# Supplementary material for: Perceptions of plastic pollution among inland fishery stakeholders in a subtropical reservoir
Source: PLoS One. 2026 Jul 9;21(7):e0353457. doi: 10.1371/journal.pone.0353457 (PMC13349089; doi:10.1371/journal.pone.0353457)
Supplement: S4 Table — Abbreviation: CF – Commercial fisher, RF – Recreational fisher, FM – Fishmongers. (DOCX) [file pone.0353457.s004.docx]

**S4 Table**: Local fishery stakeholder responses (%) regarding seasonal occurrence of plastic pollution around Nandoni Dam. Abbreviation: CF – Commercial fisher, RF – Recreational fisher, FM – Fishmongers.

| Season | CF | RF | FM |
| --- | --- | --- | --- |
| Summer | 100 | 50 | 80 |
| Winter | 0 | 20 | 0 |
| Spring | 0 | 20 | 10 |
| Throughout the year | 0 | 0 | 10 |
| Don’t know | 0 | 10 | 0 |
