# Supplementary material for: Perceptions of plastic pollution among inland fishery stakeholders in a subtropical reservoir
Source: PLoS One. 2026 Jul 9;21(7):e0353457. doi: 10.1371/journal.pone.0353457 (PMC13349089; doi:10.1371/journal.pone.0353457)
Supplement: S5 Table — Abbreviation: CF – Commercial fisher, RF – Recreational fisher, FM – Fishmongers. (DOCX) [file pone.0353457.s005.docx]

**S5 Table**: Local fishery stakeholder responses (%) to multiple questions. Abbreviation: CF – Commercial fisher, RF – Recreational fisher, FM – Fishmongers.

| Stakeholder | Yes | No |
| --- | --- | --- |
| Have you observed plastic pollution in or around Nandoni Dam? | | |
| CF | 100 | 0 |
| RF | 90 | 10 |
| FM | 100 | 0 |
| Do you think pollution in the Dam affects your income or livelihood? | | |
| CF | 80 | 20 |
| RF | 20 | 80 |
| FM | 40 | 60 |
| Have you ever received training or education on the dangers of plastic pollution and how to reduce it? | | |
| CF | 10 | 90 |
| RF | 60 | 40 |
| FM | 0 | 100 |
| At a personal level, would you implement any measures to reduce the use of plastic within the local communities around Nandoni Dam? | | |
| CF | 90 | 10 |
| RF | 90 | 10 |
| FM | 90 | 10 |
